# Therapeutic potential of Licochalcone A in dermatological diseases: from basic to clinical research

**DOI:** 10.3389/fphar.2025.1632006

**Published:** 2025-10-16

**Authors:** Deming Liu, Xue Jiang, Fujin Yang, Jingjing Zhou, Yanxi Li, Hua Yang

**Affiliations:** Chongqing Clinical Research Center for Dermatology, Chongqing Key Laboratory of Integrative Dermatology Research, Key Laboratory of External Therapies of Traditional Chinese Medicine in Eczema, Department of Dermatology, Chongqing Traditional Chinese Medicine Hospital, The First Affiliated Hospital of Chongqing College of Traditional Chinese Medicine, Chongqing, China

**Keywords:** Licochalcone A, dermatological diseases, multi-target therapy, Nrf2/HO signaling pathway, clinical translation

## Abstract

Licochalcone A (Lico-A), a flavonoid compound extracted from Glycyrrhiza uralensis, exhibiting multiple pharmacological properties including anti-inflammatory, antibacterial, antioxidant, and antitumor effects. It demonstrates significant therapeutic potential in the field of dermatological treatment. This review focuses on the efficacy of Lico-A in the treatment of acne, atopic dermatitis, rosacea, pigmentation disorders, and skin tumors. Mechanistically, Lico-A targets multiple signaling pathways such as NLRP3 inflammasome, NF-κB, PLC/ERK/STAT3, AP-1, and Nrf2/HO-1, thereby modulating inflammatory cascades, oxidative stress, melanogenesis, and tumorigenic processes. Clinical studies have also confirmed its ability to reduce inflammatory lesions, improve skin barrier function, and suppressing hyperpigmentation. However, current research is limited by geographical bias, a lack of high-quality randomized controlled trials, and reliance on preclinical models. Future studies should focus on multicenter randomized controlled trials, advanced delivery systems, and mechanistic investigations using systems biology. Addressing these gaps could establish Lico-A as a multifunctional, evidence-based dermatologic therapy for inflammatory, infectious, and neoplastic skin disorders.

## 1 Introduction

Skin diseases are the fourth leading cause of disability worldwide, affecting approximately one-third of the world’s population (Flohr and Hay, 2021; Trakatelli et al., 2023). In 2019, the burden of skin and subcutaneous diseases, measured in disability-adjusted life years, reached 42,883,695.48, underscoring their substantial impact on global health and the consequent need for effective therapeutic interventions (Yakupu et al., 2023). Current treatment modalities for skin diseases include antibiotics, glucocorticoids, immunosuppressants, and biologics. These agents are often limited by transient efficacy, high relapse rates upon discontinuation, and the emergence of drug resistance. Additionally, some medications are associated with hepatotoxicity and nephrotoxicity, highlighting the urgent demand for safer and more effective alternatives.

**FIGURE 1:**
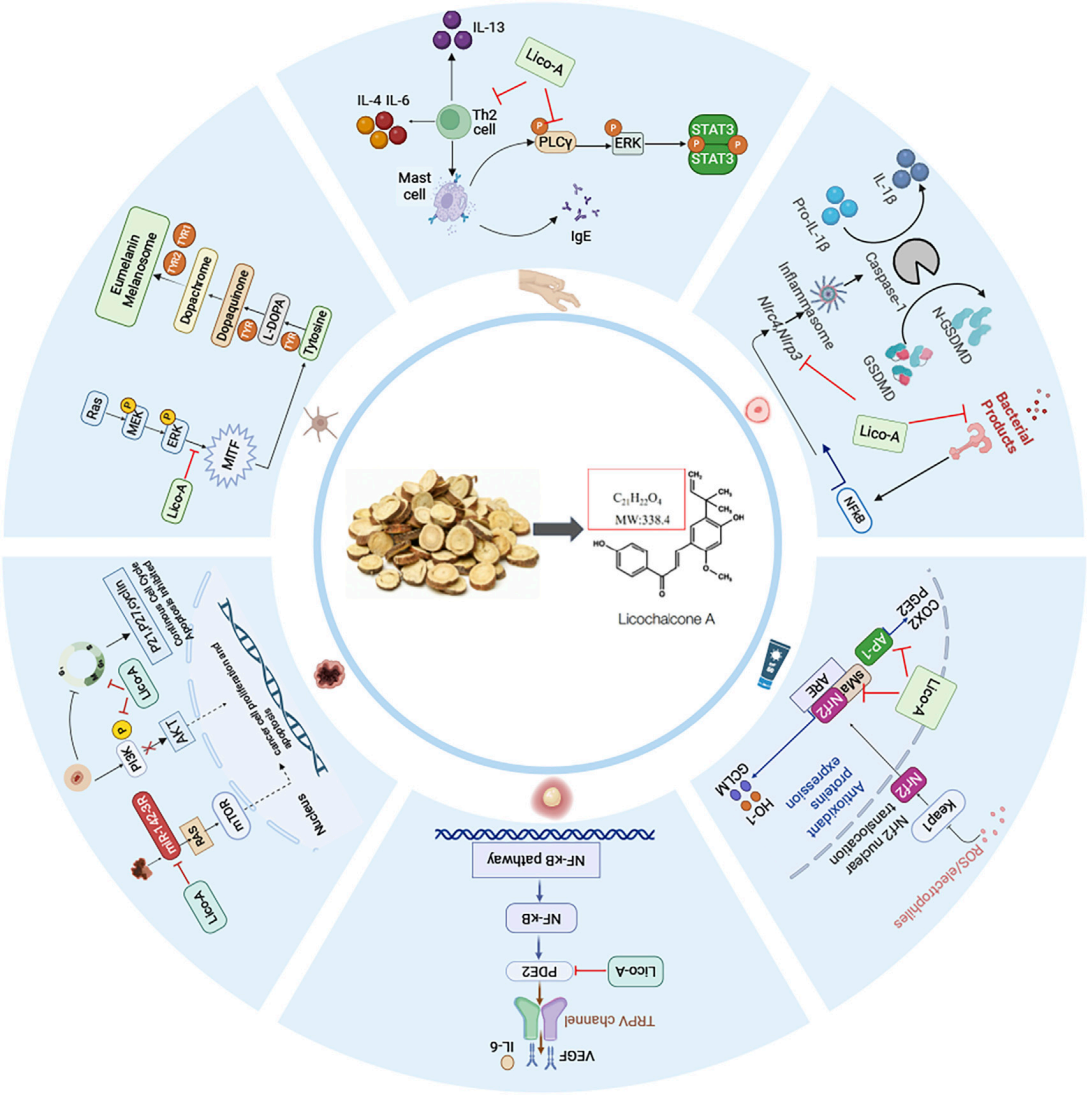
The main targeted signaling pathways and action mechanism of Lico-A in the treatment of dermatologic diseases.

Recently, plant-derived natural compounds have emerged as promising therapeutic candidates for dermatological conditions, owing to their low skin irritancy, reduced propensity for resistance development, and ability to modulate skin microbiota (Wang et al., 2025). Among these compounds, licochalcone A (Lico-A), a flavonoid isolated from the roots and stems of Glycyrrhiza uralensis Fisch., has emerged as a promising therapeutic candidate for tumors, infectious diseases, and inflammatory disorders due to its broad-spectrum pharmacological activities including anti-inflammatory, antitumor, antimicrobial, antioxidant, and neuroprotective effects (Li et al., 2024; Liu et al., 2018; Wang et al., 2024). Preliminary clinical evidence suggests that Lico-A alleviates symptoms of acne, rosacea, and atopic dermatitis (AD) while preventing disease recurrence (Olloquequi et al., 2023). (Table 1; Figure 1). Its multi-targeted mechanism of action has further fueled interest in its application for dermatological conditions.

**TABLE 1:** Clinical research and mechanism of action of Lico-A.

Disease	Drug	Type of study	Experimental model	Mode of drug administration	Dose range tested	Duration	Main findings	References
Acne	treatment group: (1) Lico-A/Salicylic acid/L-Carnitine Mixed Solution (2) Lico-A/Hydroxy-Complex 10% cream	Clinical study	91 Acne patients	topical treatment	—	8 weeks	A cosmetic regimen containing Lico-A significantly reduced the number of comedones and papules and markedly suppressed sebum secretion in mild acne	Dall‘Oglio et al. (2019)
Acne	(1) treatment group: Lico-A/decanediol/L-carnitine/1% salicylic acid moisturizer (2) control group: moisturizer vehicle	Clinical study	110 Acne patients	topical treatment	—	20 weeks	Moisturizer containing Lico-A reduced acne lesions and prevented the development of new lesions during the maintenance phase	Kulthanan et al. (2020)
Acne seborrheic dermatitis	(1) treatment group Lico-A/1,2-decanediol/L-carnitine/salicylic acid moisturizer	Clinical study	20 patients with acne or seborrheic dermatitis	topical treatment	—	8 weeks	The moisturizer containing Lico-A exhibits both sebum-suppressive and lipid-modulating effects	Wongtada et al. (2022)
Acne	(1) treatment group: adapalene gel with Lico-A/l-carnitine/1,2-decanediol moisturizers (2) control group: adapalene gel, adapalene gel with the placebo	Clinical study	120 Acne patients	topical treatment	—	8 weeks	The combined use of adapalene gel and the moisturizer containing Lico-A demonstrates effect of reducing adverse effects while enhancing therapeutic efficacy	Chularojanamontri L et al. (2016)
Acne	(1) treatment group: 7%Glycolic acid/1%salicylic acid/2%gluconolactone/0.05%Lico-A/0.1%adapalene gel cosmeceutical products (2) control group 0.1% adapalene	Clinical study	25 Acne patients	topical treatment	—	4 weeks	The Lico-A-adapalene cosmeceutical combination exhibits non-inferior therapeutic effects to adapalene alone for mild-to-moderate acne, while demonstrating superior tolerability	Kantikosum et al. (2019)
Acne	(1) treatment group Lico-A/L-carnitine/decanediol cream with PDT (2) control group: the vehicle cream with PDT	Clinical study	29 Acne patients	topical treatment	—	10 weeks	Topical application of Lico-A -based cream potentiates the therapeutic effects of photodynamic therapy on acne lesions, while concurrently mitigating post-inflammatory hyperpigmentation	Wanitphakdeedecha et al. (2020)
Acne	(1) treatment group: broad spectrum sunscreen, containing Lico-A, l-carnitine, octocrylene, butyl methoxy dibenzoylmethane, homosalate (2) control group: UV filters	Clinical study	59 Acne patients	topical treatment	—	6 weeks	Topical application of Lico-A-enhanced sunscreen demonstrates significant preventive effects on post-inflammatory hyperpigmentation following picosecond laser treatment	Puaratanaarunkon and Asawanonda., (2022)
AD	(1) treatment group Lico-A (2) control group: 1% hydrocortisone	Clinical study	55 Childhood atopic dermatitis patients	topical treatment	—	4 weeks	For atopic dermatitis management, licorice extract exhibits non-inferior therapeutic effects to hydrocortisone butyrate 0.1% cream while demonstrating comparable cutaneous tolerability	Wananukul et al. (2013)
AD	(1) treatment group Lico-A (2) control group: 1% hydrocortisone	Clinical study	26 Childhood atopic dermatitis patients	topical treatment	—	6 weeks	The effectiveness of LA lotion is equal to that of HC lotion for childhood atopic dermatitis	Udompataikul and Srisatwaja. (2011)
AD	(1) treatment group Lico-A/atty acid/glycerol/ceramide blend emulsion	Clinical study	26 Atopic dermatitis patients	topical treatment	—	12 weeks	Topical application of a Lico-A -based moisturizing cream demonstrates dual benefits in significantly preventing disease flares and preserving epidermal barrier function in atopic dermatitis management	Angelova-Fischer et al. (2018)
Facial dermatitis	(1) treatment group: moisturizer containing 4-t-butylcyclohexanol, Lico-A (2) control group 0.02% triamcinolone acetonide	Clinical study	80 Facial dermatitis patients	topical treatment	—	4 weeks	While exhibiting delayed therapeutic onset relative to triamcinolone acetonide, the Lico-A-enriched moisturizer shows clinically significant advantages in erythema reduction and stratum corneum hydration improvement in facial dermatitis patients	Boochai, et al. (2018)
Sensitive skin	(1) treatment group Lico-A/4-t-butylcyclohexanol (2) control group: 4-t-butylcyclohexanol/Lico-A/acetyl dipeptide-1 cetyl ester	Clinical study	38 Sensitive skin patients	topical treatment	—	24 h	Topical application of 4-t-butylcyclohexanol and Lico-A demonstrates synergistic efficacy in immediately alleviating neurosensory symptoms	Sulzberger et al. (2016)
Rosacea	(1) treatment group Lico-A 4-t-butylcyclohexanol	Clinical study	32 Rosacea patients	topical treatment	—	8 weeks	A Lico-A-based skincare system significantly reduces erythema intensity and improves tactile roughness in reactive skin types	Schoelermann et al. (2016)
Sensitive skin Rosacea	(1) treatment group: combination of trans-t-butylcyclohexanol/Lico-A	Clinical study	1,221 patients with Sensitive skin or Rosacea	topical treatment	—	4 weeks	The combination of trans-4-t-butylcyclohexanol and Lico-A demonstrates significant therapeutic efficacy and excellent tolerability in managing sensitive skin and rosacea	Jovanovic et al. (2017)
Acne	Lico-A	*In vivo*	P.acnes-induced Acne mice mouse	local injection	1.25%–2.5%	24 h	Lico-A inhibits NLRP3 inflammasome, thereby suppressing inflammation induced by Propionibacterium acnes	Yang et al. (2018)
AD	Lico-A 1% sodium pentobarbital	*In vivo*	IgE-mediated allergic mouse models	gastric lavage	20–80 mg/kg	30 min	Licorice chalcone A reduces inflammation in mice, restores hypothermia caused by allergies, and reduces tumor necrosis factor and monocyte chemotactic protein	Shu et al. (2022)
Photosensitivity disorders	Lico-A	*In vitro*	HaCaT cells	-	1.25–10 μM	1 h	Lico-A reduces UVR-induced skin tissue damage by targeting the AP-1 transcription factor, inhibits UVR-induced COX-2 expression and PGE2 production	Song et al. (2015)
Photosensitivity disorders	Lico-A	*In vitro*	Primary human dermal fibroblasts	-	0.25–2 μM	24 h	Lico-A could activate the transcription factor Nrf2, inhibit the generation of ROS induced by visible light, reduce the cellular damage caused by oxidative stress, and reduce the consumption of carotenoids in the skin by visible light	Mann et al. (2020)
Photosensitivity disorders	Lico-A	*In vitro*	Primary human fibroblasts	-	0.25–16 μM	6h–24 h	Lico-A induces nuclear translocation of Nrf2 and upregulates the expression of HO-1 and GCLM.	Kühnl et al. (2015)
Pigmented skin diseases	Lico-A	*In vitro*	B16 cells	-	6.5–104 mg/mL	24 h	Lico-A inhibits melanin content in B16 cells by phosphorylating ERK, downregulating MITF, and reducing tyrosinase activity	Hong et al. (2018)
OSCC	Lico-A	*In vitro*	HN22 and HSC4 cell lines	-	0.25–1, 2 μM	48 h	Licorice chalcone A significantly inhibits HN22 and HSC4 cells	Cho et al. (2014)
OSCC	Lico-A	*In vitro*	SCC4 and CAL-27 cell lines	-	25–100 μM	48 h	Lico-A inhibits SCC4 and CAL-27 cell proliferation	Hao et al. (2019)
Melanoma	Lico-A	*In vitro*	A375 and B16 melanoma cells	-	5–20 μM	24h–72 h	Lico-A activates miR-142-3p, promotes the expression of Ras homolog protein, activates the mTOR signaling pathway, inhibits the proliferation of A375 and B16 melanoma cells, and induces apoptosis	Zhang et al. (2020)
Microsporum canis	Lico-A	*In vitro*	Microsporum canis ATCC 36299	-	0.25–1064 μM	5–25 h	Lico-A inhibits fungal growth by suppressing ergosterol biosynthesis, reducing ATPase activity to impair energy metabolism, and inhibiting ROS-induced oxidative stress	Sun et al. (2025)
Aspergillus fumigatus Keratitis	Lico-A	*In vivo*	Aspergillus fumigatus Keratitis mice		12–60 μM	24h–72 h	Lico-A inhibits ergosterol biosynthesis, disrupts cell membrane integrity, and suppresses the growth of Aspergillus fumigatus	Tian et al. (2024)
Leishmaniasis	Lico-A	*In vitro*	leishmania	-	5–10 μg	20 h	Lico-A can inhibit the growth of Leishmania and Donovaniasis parasites	Chen et al. (2014)

To comprehensively evaluate the therapeutic potential and mechanistic basis of Lico-A in dermatology, we conducted a systematic review of clinical and preclinical studies published in the past decade, sourced from PubMed and Embase. Search terms included “licorice,” “Lico-A,” “skin diseases,” “inflammation,” and “tumors,” among others. This review summarizes the latest advances in the pharmacological research and clinical applications of Lico-A in dermatology, systematically analyzes the advantages and disadvantages of Lico-A in the treatment of various skin diseases and explores future research directions such as its multi-target mechanism and development of novel drug delivery systems.

## 2 Application of Lico-A in inflammatory dermatological diseases

### 2.1 Acne vulgaris

Acne vulgaris represents a chronic inflammatory condition of the pilosebaceous unit (Vasam et al., 2023), primarily associated with hyperseborrhea, Cutibacterium acnes colonization, inflammatory cascades, and follicular hyperkeratinization (Reynolds et al., 2024). Lico-A can modulate key pathogenic pathways of acne through multi-target mechanisms, demonstrating significant clinical therapeutic potential. Multiple clinical studies have used topical formulations containing Lico-A for acne patients. Results indicate that these formulations significantly suppress sebum secretion and reduce the formation of comedones, inflammatory papules, and non-inflammatory papules, preventing disease progression (Wongtada et al., 2022; Kulthanan et al., 2020; Dall'Oglio et al., 2019). A randomized controlled trial in an Asian population demonstrated that a moisturizer containing 0.5% Lico-A combined with 0.1% adapalene gel significantly reduced inflammatory and total lesion counts in mild-to-moderate acne, without the initial inflammatory exacerbation commonly observed with retinoid monotherapy (Chularojanamontri et al., 2016). This study confirmed that Lico-A not only mitigates the skin irritation associated with retinoids but also prevents the initial acne flare-up, thereby improving patient adherence.

Additionally, Lico-A has shown beneficial effects when combined with light-based therapies. The combination of Lico-A moisturizer and photodynamic therapy significantly reduced lesion counts in moderate-to-severe acne, enhanced therapeutic efficacy, minimized adverse effects, and markedly decreased post-inflammatory hyperpigmentation (Wanitphakdeedecha et al., 2020). Puaratanaarunkon T’s study further demonstrated that Lico-A cream, when used with picosecond laser treatment, reduced inflammatory papules and hyperpigmentation in acne patients (Puaratanaarunkon and Asawanonda, 2022), outperforming a control group using only conventional ultraviolet filters. These findings further support the safety and efficacy of Lico-A in combination with photodynamic therapy for moderate-to-severe acne.

Further mechanism studies confirmed that Lico-A effectively inhibits P. acnes-induced NLRP3 inflammasome activation, reduces the production of mitochondrial reactive oxygen species (ROS), and subsequently blocks caspase-1 and IL-1β secretion. In murine models, topical application of Lico-A significantly alleviated P. acnes-induced skin inflammation, erythema, and inflammatory cell infiltration, with efficacy comparable to benzoyl peroxide, a clinically standard treatment. Furthermore, Lico-A at concentrations of 10 μM and 20 μM exhibited direct antibacterial activity against P. acnes, reducing its abnormal follicular colonization (Yang et al., 2018). These findings provide a theoretical foundation for the development of Lico-A as a novel anti-acne therapeutic agent.

In summary, Lico-A demonstrates significant efficacy in preventing and treating acne by inhibiting NLRP3 inflammasome activation, reducing mitochondrial ROS production, and exerting direct antibacterial effects. However, current clinical evidence remains limited to small-scale trials with notable geographic bias and lacks long-term safety data. Future research should prioritize larger, high-quality clinical studies and broader mechanistic investigations, including Lico-A’s modulation of C. acnes biofilm formation, sebaceous gland activity, and inflammatory crosstalk.

### 2.2 Atopic dermatitis

AD is a common chronic relapsing inflammatory disease in dermatology, with type 2 inflammation and barrier dysfunction as important pathogenic mechanisms (Kellogg and Smogorzewski, 2023).The significant inhibition of Th2 cytokines by Lico-A also makes it a potential treatment for AD (Huang et al., 2019; Sherwani et al., 2024).

Multiple clinical studies have demonstrated the efficacy of Lico-A in alleviating AD. Research by Wananukul S et al. revealed that adult AD patients applying Lico-A formulations twice daily for 8 weeks exhibited significant reductions in SCORAD scores, with comparable efficacy to 1% hydrocortisone. Lico-A demonstrated superior outcomes in reducing transepidermal water loss (TEWL), improving skin barrier function, and lowering relapse rates. These findings align with Udompataikul M’s pediatric AD study, collectively indicating that Lico-A not only mitigates AD symptoms but also reduces corticosteroid dependency (Wananukul et al., 2023; Udompataikul and Srisatwaja, 2011). A 12-week double-blind randomized split-face relapse prevention trial further showed that Lico-A emollients reduced relapse rates by 60% in adult AD patients versus controls, significantly alleviating recurrent rash and pruritus while decreasing *Staphylococcus aureus* colonization and maintaining skin barrier homeostasis, highlighting Lico-A’s long-term therapeutic advantages (Angelova-Fischer et al., 2018). Additionally, a randomized prospective investigator-blinded study confirmed that Lico-A emollients exhibited superior sustained anti-inflammatory effects and skin hydration compared to 0.02% triamcinolone cream in facial AD, suggesting its potential for treating region-specific AD (Boonchai et al., 2018). Mechanistically, beyond Th2 cytokine modulation, Shu et al. (2022) demonstrated in an IgE-mediated murine allergy model that Lico-A suppresses PLC/ERK/STAT3 signaling, thereby inhibiting mast cell activation and reducing tumor necrosis factor-α (TNF-α) and MCP-1 release to attenuate inflammatory responses—further elucidating its anti-allergic mechanisms (Shu et al., 2022).

In conclusion, Lico-A alleviates AD by suppressing mast cell activation through inhibition of Th2 cytokines and the PLC/ERK/STAT3 signaling pathway, thereby reducing TNF-α and MCP-1 release. However, current clinical studies on Lico-A for AD are outdated, with a lack of recent trials. Moreover, existing mechanistic research remains limited, focusing primarily on its anti-inflammatory effects. Future studies should prioritize high-quality randomized controlled trial (RCT) and broader mechanistic investigations, including Lico-A’s effects on skin barrier function and neuroimmune interactions.

### 2.3 Rosacea and sensitive skin

Rosacea and sensitive skin are common chronic inflammatory dermatoses whose pathogenesis involves skin barrier dysfunction, neurovascular dysregulation, and immune-inflammatory responses (Geng et al., 2024; Sharma et al., 2022; Goh et al., 2023). In recent years, Lico-A has demonstrated unique therapeutic potential for these conditions due to its multi-target pharmacological activities.

Sulzberger et al. (2016); Kolbe et al. (2006) established post-shaving skin irritation models to evaluate the anti-irritant and skin-soothing effects of Lico-A-containing formulations. Their findings revealed that these formulations promoted erythema resolution and significantly reduced inflammatory responses within 24 h post-shaving, demonstrating notable skin-calming properties (Sulzberger et al., 2016; Kolbe et al., 2006). Schoelermann et al. (2016) conducted a clinical study in which skincare products containing Lico-A were applied to patients with mild-to-moderate rosacea. After 4 weeks, the treatment led to significant improvements in facial erythema, telangiectasia, roughness, and tightness, accompanied by reduced TEWL, increased skin hydration, and enhanced barrier function (Schoelermann et al., 2016). To further validate these observations, a large-scale study involving 1,221 patients with sensitive skin and rosacea was conducted to assess the efficacy and tolerability of Lico-A-containing cosmetics. Consistent with previous findings, the results confirmed not only clinical symptom relief but also excellent tolerability with no significant adverse effects (Jovanovic et al., 2017).

Mechanistic studies revealed that even at low concentrations (1.5 μM), Lico-A markedly suppresses TNF-α-induced NF-κB pathway activation and reduces the secretion of inflammatory mediators such as prostaglandin E2 (PGE2). This mechanism is particularly critical, as PGE2 not only directly participates in inflammatory responses but also sensitizes sensory neurons via TRPV1 channel activation, thereby inducing the release of TNF-α, interleukin-6, and vascular endothelial growth factor, which exacerbate vasodilation and non-neurogenic inflammation (Sulzberger et al., 2016). By disrupting this vicious cycle, Lico-A alleviates neurovascular hyperreactivity at its source.

In summary, Lico-A alleviates erythema and dryness in rosacea and sensitive skin by suppressing neurovascular inflammation through inhibition of the NF-κB pathway, thereby reducing PGE2 secretion. However, current clinical studies primarily evaluate Lico-A in combination formulations, with a lack of research on its efficacy as a standalone treatment. Additionally, mechanistic investigations remain limited. Future research should prioritize high-quality RCTs focusing on Lico-A monotherapy, along with further exploration of its mechanisms in neurovascular inflammation regulation.

### 2.4 Photodermatoses

Photodermatoses are a group of olar radiation-induced or exacerbated cutaneous disorders, including chronic actinic dermatitis, juvenile spring eruption, actinic prurigo and so on (Burfield et al., 2023). Ultraviolet radiation (UVR) and visible light (VIS) serve as primary etiological factors for these photosensitivity disorders (McDonald et al., 2023), inducing pathological consequences through DNA damage, oxidative stress, and inflammatory cascades (Tang et al., 2024). Lico-A has emerged as a promising candidate for photoprotection and photo-dermatoses management due to its dual anti-inflammatory and antioxidant properties.

Molecular studies have elucidated Lico-A’s mechanisms of action. Song et al. (2015) demonstrated that Lico-A specifically targets the AP-1 transcription factor, significantly suppressing UVR-induced COX-2 expression and PGE2 production. Importantly, this mechanism not only attenuates photoinflammatory responses but also disrupts the vicious cycle between inflammation and oxidative stress (Song et al., 2015). Mann et al. (2020) established a high-energy visible light-induced oxidative stress model, confirming that Lico-A’s photoprotective effects involve activation of the nuclear factor erythroid-2-related factor 2 (Nrf2) pathway. This activation mitigates ROS-mediated lipid peroxidation, DNA/protein damage, and prevents VIS-induced cutaneous carotenoid depletion (Mann et al., 2020). Kühnl et al. (2015) further elucidated the antioxidant mechanism of Lico-A, demonstrating its capacity to induce Nrf2 nuclear translocation and subsequent upregulation of critical antioxidant enzymes, including heme oxygenase-1 (HO-1) and glutamate-cysteine ligase (GCLM). Mechanistically, the catalytic products of HO-1 (biliverdin and carbon monoxide) exhibit synergistic antioxidant and anti-inflammatory properties, whereas GCLM maintains cellular redox homeostasis by regulating glutathione biosynthesis (Kühnl et al., 2015). These molecular mechanisms were clinically validated by Lim et al., 2022 demonstrating that Lico-A-containing formulations significantly reduce UVR/VIS-induced ROS generation while improving clinical manifestations including erythema and hyperpigmentation (Lim et al., 2022).

In conclusion, Lico-A exerts therapeutic effects on photodermatoses through dual modulation of Nrf2 activation and AP-1 suppression. However, current research has primarily focused on elucidating its molecular mechanisms, with limited clinical validation. Future studies should prioritize well-designed clinical trials to evaluate the therapeutic potential of Lico-A in photosensitive disorders, thereby providing an evidence-based foundation for its clinical application in photodermatoses.

## 3 Application of Lico-A in pigmented skin diseases

Excessive melanin production represents the central pathogenic mechanism in cutaneous pigmentation disorders. The microphthalmia-associated transcription factor (MITF) serves as the master regulator of melanogenesis, controlling expression of key enzymes including tyrosinase (TYR), tyrosinase-related protein 1 and tyrosinase-related protein 2. TYR is the key enzyme in melanin synthesis (Qu et al., 2023). And natural skin-lightening agents exert depigmenting effects through multiple pathways, including TYR inhibition, suppression of MAPK signaling, and interference with melanosome transport (Liu et al., 2024).

Hong et al. (2018) applied different concentrations (6.5–104 mg/mL) of Lico-A on melanoma cells B16 to evaluate the role of Lico-A in pigmentary diseases. The results showed that after 24 h, the amount of melanin in B16 cells was significantly reduced and the tyrosinase activity was significantly attenuated. Mechanistically, Lico-A activates ERK phosphorylation, leading to MITF downregulation and consequent tyrosinase inhibition (Hong et al., 2018). Studies indicate that Lico-A exerts its skin-whitening effects primarily through suppression of MITF expression rather than via direct inhibition of tyrosinase enzymatic activity, which is consistent with the findings reported by Jung et al. (2025). The depigmentation mechanism of Lico-A circumvents the pigmentary rebound effect associated with direct tyrosinase inhibition, thereby conferring therapeutic advantages for treating pigmentary disorders.

Wang et al. (2021) developed a Lico-A-loaded glycyrrhiza acid (GA + Lico-A) micelles with enhanced epidermal delivery and depigmentation efficacy, while systematically evaluating the depigmentation efficacy of GA + Lico-A micelles through both *in vitro* and *in vivo* experiments. The results demonstrated that GA + Lico-A micelles exhibited superior tyrosinase inhibitory effects and melanin-reducing capacity in B16 cells compared to Lico-A alone. Furthermore, animal studies demonstrated that 12-day consecutive topical application of both (GA + Lico-A) micelles and Lico-A significantly attenuated UVB-induced hyperpigmentation in murine models, within MAPK/ERK pathway distribution patterns correlating precisely with areas of decreased tyrosinase activity (Wang et al., 2025).

Further supporting evidence emerged from a comprehensive comparative analysis of flavonoid compounds conducted by Fu et al., 2005 which identified Lico-A as a particularly potent tyrosinase inhibitor among five tested flavonoids. Structure-activity relationship studies highlighted the critical importance of hydroxyl group positioning for optimal enzyme inhibition, providing molecular-level insights into Lico-A’s depigmentation mechanism (Fu et al., 2005).

While these preclinical studies establish Lico-A as a promising multi-target depigmenting agent through MITF downregulation and tyrosinase inhibition, clinical study remains limited (Figure 1). Further investigation is required to validate its efficacy, safety profile, and precise mechanisms of action in human pigmentation disorders.

## 4 Application of Lico-A in skin tumors

Cancer represents a group of malignant diseases that pose serious threats to human health and life. Its hallmark characteristics include rapid proliferation, invasion, and migration of abnormal cells (White et al., 2024). Lico-A has been shown to significantly inhibit proliferation and promote apoptosis in various cancer cell types. In dermatological oncology, Lico-A demonstrates particular therapeutic potential for oral squamous cell carcinoma (OSCC) and melanoma.

Lico-A exhibits distinct anti-OSCC mechanisms across different SCC models. In SCC-25 cells, Lico-A inhibits proliferation by inducing cell cycle arrest at S and G2/M phases. Conversely, in HN22 and HSC4 cell lines, Lico-A exerts its anticancer effects through suppression of specificity protein 1 (Sp1) and its downstream targets, including p27, p21, cyclin D1 and surviving (Cho et al., 2014). Further investigations by Hao et al. (2019) revealed that Lico-A exerts antiproliferative effects on SCC4 and CAL-27 OSCC cell lines through modulation of the PI3K/AKT signaling pathway. *In vivo* studies showed that Lico-A administration significantly reduced tumor burden in murine models of squamous cell carcinoma, concomitant with downregulation of metastasis-associated markers MMP-2 and MMP-9 (Hao et al., 2019). In a parallel study focusing on melanoma, Zhang et al. (2020) demonstrated that Lico-A enhances the expression of Ras homologous protein enriched in the brain by activating miR-142-3p, thereby activating the mTOR signaling pathway, inhibiting proliferation, reducing melanin production, and inducing apoptosis in A375 and B16 melanoma cells (Zhang et al., 2020).

In conclusion, Lico-A demonstrates multi-target anticancer potential in oral squamous cell carcinoma and melanoma through Sp1 suppression, PI3K/AKT pathway blockade, and mTOR pathway modulation (Figure 1). However, its mechanistic variability across models underscores the need for: (1) unified mechanistic studies, (2) advanced *in vivo* models, (3) early-phase clinical trials to evaluate human safety and efficacy.

## 5 Application of Lico-A in infections

Microbial infections represent a major etiological factor in dermatological pathologies (Hansen et al., 2022). Lico-A shows broad-spectrum antimicrobial activity against fungi, bacteria, and parasites via multi-target mechanisms.

### 5.1 Antifungal activity and mechanisms

Lico-A exhibits potent inhibitory activity against Microsporum canis (MIC = 4 μg/mL), with activity significantly superior to that of fluconazole compared to fluconazole (MIC = 64 μg/mL). Its multifaceted mechanism involves ergosterol biosynthesis inhibition-mediated membrane disruption, ATPase activity reduction impairing energy metabolism, and ROS-induced apoptotic oxidative stress. Transcriptomic analyses further reveal that Lico-A modulates critical pathways including cell wall biosynthesis, tricarboxylic acid cycle, and oxidative phosphorylation, resulting in comprehensive fungal growth suppression (Sun et al., 2025). Against *Candida* albicans and Aspergillus fumigatus, Lico-A inhibits biofilm formation, reduces host cell adhesion, and exerts direct killing effects through hyphal deformation and mitochondrial damage, while activating the host Nrf2/HO-1 pathway to mitigate inflammatory responses (Tian et al., 2024; Li et al., 2022).

### 5.2 Antibacterial and wound healing properties

Beyond its antifungal effects, Lico-A demonstrates substantial antibacterial activity against *Escherichia coli* and *Staphylococcus aureus*. When incorporated into chitosan-hyaluronic acid hydrogels, Lico-A maintains sustained-release properties and antimicrobial efficacy while accelerating wound healing through attenuation of inflammation, promotion of collagen synthesis, and stimulation of angiogenesis (Hou et al., 2024; Qu et al., 2023).

### 5.3 Antiparasitic efficacy

Lico-A displays potent activity against both promastigote and amastigote forms of Leishmania species. Experimental evidence demonstrates its capacity to significantly reduce infection rates in macrophages and U937 cells, while *in vivo* administration markedly decreases parasitic burden in hepatic and splenic tissues and prevents cutaneous lesion development in murine models (Chen et al., 1993).

In summary, Lico-A represents a promising antimicrobial agent with broad-spectrum antibacterial activity that exhibits significant antibacterial effects against fungal, bacterial, and parasitic pathogens through membrane disruption, bioenergetic interference, and immune regulation. Future investigations should focus on delivery system optimization to enhance bioavailability and targeting specificity, potentially positioning Lico-A as a next-generation antimicrobial for dermatological applications.

## 6 Discussion

Lico-A has demonstrated significant therapeutic potential across multiple dermatological conditions including acne vulgaris, AD, and rosacea, with mechanisms of action involving anti-inflammatory, antimicrobial, antioxidant, and antitumor. Clinical studies have confirmed its efficacy in reducing acne lesions, improving skin barrier function, and alleviating erythema, while exhibiting superior tolerability compared to conventional therapies such as corticosteroids and retinoids. These properties have positioned Lico-A as a compound of particular interest to dermatology specialists. However, several limitations persist in current research.

A critical issue is the geographical restriction of clinical evidence, with most studies conducted in Asian populations, raising questions about the generalizability of findings to other ethnic groups. The field also suffers from a paucity of high-quality randomized controlled trials, as existing clinical data primarily derive from small-scale, short-term studies with limited sample sizes. This gap is particularly evident in long-term safety assessments and relapse prevention studies. Furthermore, mechanistic understanding of Lico-A remains largely dependent on preclinical models including *in vitro* cell cultures and murine studies, which may not fully recapitulate the complexity of human skin pathophysiology. The therapeutic performance of Lico-A is additionally influenced by delivery system selection. While topical formulations such as creams and moisturizers minimize systemic side effects, their efficacy is constrained by poor skin penetration and short residence time. Microemulsion-based systems can enhance epidermal delivery efficiency but face challenges in stability and large-scale production. Hydrogels offer sustained antimicrobial release and wound healing benefits, though their high water content may compromise drug loading capacity.

To advance the clinical translation of Lico-A in dermatology, future investigations should prioritize global multicenter randomized controlled trials incorporating diverse patient populations, long-term follow-up, and rigorous methodological standards. Concurrent optimization of Lico-A formulations - including monotherapy trials and advanced delivery systems such as nanoparticles - is necessary to clarify its standalone therapeutic profile. A paradigm shift toward systems biology and network pharmacology approaches will be crucial to comprehensively elucidate Lico-A’s pleiotropic mechanisms and refine clinical applications. Given the multifactorial etiology of many dermatoses, integrating Lico-A into multimodal combination therapies shows particular promise. Such combinatorial strategies may not only amplify therapeutic outcomes but also mitigate resistance development, a persistent challenge in chronic dermatological management. Finally, clinical implementation must prioritize safety and efficacy evaluations across special populations, particularly vulnerable groups including pediatric and geriatric patients, where careful benefit-risk assessment remains essential.

In summary, realizing the full therapeutic potential of Lico-A in dermatology requires an integrated approach combining systems biology, advanced drug delivery technologies, and innovative combination therapies. Meanwhile, standardized regulatory frameworks and enhanced strategic industry partnerships should be pursued to facilitate commercialization, establishing a clear translational pathway from mechanistic insights to clinically viable therapies. By addressing current gaps, Lico-A may emerge as a cornerstone treatment for diverse dermatological conditions.

## Abbreviations

A, Licochalcone A Lico; ROS, Reactive oxygen species; AD, Atopic dermatitis; RCT, Randomized controlled trial; TEWL, Transepidermal water loss; PGE2, Prostaglandin E2; TNF-α, Tumor necrosis factor-α; UVR, Ultraviolet radiation; VIS, Visible light; Nrf2, Nuclear factor erythroid-2-related factor 2; HO-1, Heme oxygenase-1; GCLM, Glutamate-cysteine ligase; MITF, Microphthalmia-associated transcription factor; TYR, Tyrosinase; GA + Lico-A, Lico-A-loaded glycyrrhiza acid; OSCC, squamous cell carcinoma.

## References

[B1] Angelova-Fischer I. Rippke F Fau - Richter D. Richter D Fau - Filbry A. Filbry A Fau - Arrowitz C. Arrowitz C Fau - Weber T. Weber T. F. A. U. (2018). Stand-alone emollient treatment reduces flares after discontinuation of topical steroid treatment in atopic dermatitis: a double-blind, randomized, vehicle-controlled, left-right comparison study. Acta Derm. Venereol. 98 (5), 517–523. 10.2340/00015555-2882 29335742

[B2] Boonchai W. Varothai S. Winayanuwattikun W. Phaitoonvatanakij S. Chaweekulrat P. Kasemsarn P. (2018). Randomized investigator-blinded comparative study of moisturizer containing 4-t-butylcyclohexanol and licochalcone A *versus* 0.02% triamcinolone acetonide cream in facial dermatitis. J. Cosmet. Dermatol 17 (6), 1130–1135. 10.1111/jocd.12499 29411520

[B3] Burfield L. Rutter K. J. Thompson B. Marjanovic E. J. Neale R. E. Rhodes L. A.-O. (2023). Systematic review of the prevalence and incidence of the photodermatoses with meta-analysis of the prevalence of polymorphic light eruption. J. Eur. Acad. Dermatol Venereol. 37 (3), 511–520. 10.1111/jdv.18772 36433668

[B4] Chen M. Christensen Sb Fau - Blom J. Blom J Fau - Lemmich E. Lemmich E Fau - Nadelmann L. Nadelmann L Fau - Fich K. Fich K Fau - Theander T. G. (1993). Licochalcone A, a novel antiparasitic agent with potent activity against human pathogenic protozoan species of leishmania. Antimicrob. Agents Chemother. 37 (12), 2550–2556. 10.1128/AAC.37.12.2550 8109916 PMC192736

[B5] Cho J. J. Chae J. I. Yoon G. Kim K. H. Cho J. H. Cho S. S. (2014). Licochalcone A, a natural chalconoid isolated from Glycyrrhiza inflata root, induces apoptosis *via* Sp1 and Sp1 regulatory proteins in oral squamous cell carcinoma. Int. J. Oncol. 45 (2), 667–674. 10.3892/ijo.2014.2461 24858379

[B6] Chularojanamontri L. Tuchinda P. Kulthanan K. Varothai S. Winayanuwattikun W. (2016). A double-blinded, randomized, vehicle-controlled study to access skin tolerability and efficacy of an anti-inflammatory moisturizer in treatment of acne with 0.1% adapalene gel. J. Dermatol. Treat. 27 (2), 140–145. 10.3109/09546634.2015.1079298 26293170

[B7] Dall'oglio F. Fabbrocini G. Tedeschi A. Donnarumma M. A.-O. Chiodini P. Micali G. A.-O. (2019). Licochalcone A in combination with salicylic acid as fluid based and hydroxy-complex 10% cream for the treatment of mild acne: A multicenter prospective trial. Clin. Cosmet. Investig. Dermatol 12, 961–967. 10.2147/CCID.S206935 32099436 PMC6997230

[B8] Flohr C. A.-O. Hay R. A.-O. (2021). Putting the burden of skin diseases on the global map. Br. J. Dermatol 184 (2), 189–190. 10.1111/bjd.19704 33544440

[B9] Fu B. Li H Fau - Wang X. Wang X Fau - Lee F. S. C. Lee F. S. F. A. U.-C. U. I. S. Cui S. (2005). Isolation and identification of flavonoids in licorice and a study of their inhibitory effects on tyrosinase. J. Agric. Food Chem. 53 (19), 7408–7414. 10.1021/jf051258h 16159166

[B10] Geng R. A.-O. X. Bourkas A. N. Mufti A. A.-O. Sibbald R. A.-O. (2024). Rosacea: pathogenesis and therapeutic correlates. J. Cutan. Med. Surg. 28 (2), 178–189. 10.1177/12034754241229365 38450615 PMC11015710

[B11] Goh C. L. Wu Y. Welsh B. Abad-Casintahan M. F. Tseng C. J. Sharad J. A.-O. (2023). Expert consensus on holistic skin care routine: focus on acne, rosacea, atopic dermatitis, and sensitive skin syndrome. J. Cosmet. Dermatol 22 (1), 45–54. 10.1111/jocd.15519 36409588

[B12] Hansen I. Augustin M. SchäFER I. Mohr N. (2022). Epidemiology of skin diseases in Germany: systematic review of the current state of research - part 3: infectious skin diseases. J. Dtsch. Dermatol Ges. 20 (5), 589–595. 10.1111/ddg.14702 35384269

[B13] Hao Y. Zhang C. Sun Y. Xu H. (2019). Licochalcone A inhibits cell proliferation, migration, and invasion through regulating the PI3K/AKT signaling pathway in oral squamous cell carcinoma. Onco Targets Ther. 12, 4427–4435. 10.2147/OTT.S201728 31239711 PMC6556467

[B14] Hong J. H. Cao S. W. Xiang S. J. Ruan S. F. An B. C. Wang Z. X. (2018). Glycyrrhiza flavonoids and its major component, licochalcone A, inhibit melanogenesis through MAPK/ERK pathway by activating ERK phosphorylation. J. Dermatol Sci. S0923- 1811 (18), 30203–2. 10.1016/2018.04.016 29730172

[B15] Hou Z. Wang Y. Chen S. Luo Z. Liu Y. (2024). Licochalcone A loaded multifunctional chitosan hyaluronic acid hydrogel with antibacterial and inflammatory regulating effects to promote wound healing. Int. J. Biol. Macromol. 283 (Pt 1), 137458. 10.1016/j.ijbiomac.2024.137458 39528175

[B16] Huang W. A.-O. Liu C. Y. Shen S. C. Chen L. C. Yeh K. W. Liu S. H. (2019). Protective effects of licochalcone A improve airway hyper-responsiveness and oxidative stress in a mouse model of asthma. Cells 8 (6), 617. 10.3390/cells8060617 31226782 PMC6628120

[B17] Jovanovic Z. Fau - Angabini N. Angabini N Fau - Ehlen S. Ehlen S Fau - Mokos Z. B. Mokos Zb Fau - Subotic M. Subotic M Fau - Neufang G. (2017). Efficacy and tolerability of a cosmetic skin care product with Trans-4-t-butylcyclohexanol and licochalcone A in subjects with sensitive skin prone to redness and rosacea. J. Drugs Dermatol 16 (6), 605–610. 10.1080/14764172.2017.1400168 28686779

[B18] Jung J. Y. Jeong H. J. Han G. A.-O. X. (2025). Antimelanogenic effect of fermented licorice water extract on murine melanoma B16F10 cells. Food Sci. Biotechnol. 34 (11), 2571–2580. 10.1007/s10068-025-01878-z 40492052 PMC12145374

[B100] Kantikosum K. Chongpison Y. Chottawornsak N. Asawanonda P. (2019). The efficacy of glycolic acid, salicylic acid, gluconolactone, and licochalcone a combined with 0.1% adapalene vs adapalene monotherapy in mild-to-moderate acne vulgaris: a double-blinded within-person comparative study. Clin. Cosmet. Investig. Dermatol. 12 151–161. 10.2147/CCID.S193730 30858720 PMC6386354

[B19] Kellogg C. Smogorzewski J. (2023). Update on atopic dermatitis. Adv. Pediatr. 70 (1), 157–170. 10.1016/j.yapd.2023.03.006 37422293

[B20] Kolbe L. Immeyer J Fau - Batzer J. Batzer J Fau - Wensorra U. Wensorra U Fau - Tom Dieck K. Tom Dieck K Fau - Mundt C. Mundt C Fau - Wolber R. (2006). Anti-inflammatory efficacy of licochalcone A: correlation of clinical potency and *in vitro* effects. Arch. Dermtol Res. 298 (1), 23–30. 10.1007/s00403-006-0654-4 16552540

[B21] Kühnl J. Roggenkamp D Fau - Gehrke S. A. Gehrke Sa Fau - StäB F. StäB F Fau - Wenck H. Wenck H Fau - Kolbe L. Kolbe L Fau - Neufang G. (2015). Licochalcone A activates Nrf2 *in vitro* and contributes to licorice extract-induced lowered cutaneous oxidative stress *in vivo* . Exp. Dermatol 24 (1), 42–47. 10.1111/exd.12588 25381913

[B22] Kulthanan K. A.-O. X. Trakanwittayarak S. A.-O. Tuchinda P. A.-O. Chularojanamontri L. A.-O. Limphoka P. A.-O. Varothai S. A.-O. A. (2020). A double-blinded, randomized, vehicle-controlled study of the efficacy of moisturizer containing licochalcone A, decanediol, L-Carnitine, and salicylic acid for prevention of acne relapse in Asian population. Biomed. Res. Int. 2020, 2857812. 10.1155/2020/2857812 33150170 PMC7603542

[B23] Li M. T. Xie L. Jiang H. M. Huang Q. Tong R. S. Li X. (2022). Role of Licochalcone A in potential pharmacological therapy: a review. Front. Pharmacol. 13, 878776. 10.3389/fphar.2022.878776 35677438 PMC9168596

[B24] Li W. Yin Y. Li T. Wang Y. Shi W. (2024). Licochalcone A protects vaginal epithelial cells against Candida albicans infection Via the TLR4/NF-κB signaling pathway (2024). J. Microbiol. 62 (7), 10.1007/s12275-024-00134-z 38819759

[B25] Lim H. W. Kohli I. Ruvolo E. Kolbe L. Hamzavi I. H. (2022). Impact of visible light on skin health: the role of antioxidants and free radical quenchers in skin protection. J. Am. Acad. Dermatol 86 (3), S27–S37. 10.1016/j.jaad.2021.12.024 34942294

[B26] Liu F. A.-O. Qu L. Li H. He J. Wang L. Fang Y. (2022). Advances in biomedical functions of natural whitening substances in the treatment of skin pigmentation diseases. Pharmaceutics 14 (11), 2308. 10.3390/pharmaceutics14112308 36365128 PMC9697978

[B27] Liu M. Du Y. Gao D. (2024). Licochalcone A: a review of its pharmacology activities and molecular mechanisms. Front. Pharmacol. 15, 1453426. 10.3389/fphar.2024.1453426 39188947 PMC11345200

[B28] Mann T. Eggers K. Rippke F. Tesch M. Buerger A. Darvin M. E. (2020). High-energy visible light at ambient doses and intensities induces oxidative stress of skin-protective effects of the antioxidant and Nrf2 inducer Licochalcone A *in vitro* and *in vivo* . Photodermatol. Photo 36 (2), 135–144. 10.1111/phpp.12523 31661571 PMC7078816

[B29] Mcdonald K. A.-O. Lytvyn Y. Mufti A. Chan A. W. Rosen C. F. (2023). Review on photoprotection: a clinician's guide to the ingredients, characteristics, adverse effects, and disease-specific benefits of chemical and physical sunscreen compounds. Arch. dermatol Res. 315 (4), 735–749. 10.1007/s00403-022-02483-4 36443500

[B30] Olloquequi J. Ettcheto M. A.-O. Cano A. Fortuna A. A.-O. Bicker J. A.-O. SáNCHEZ-Lopez E. A.-O. X. (2023). Licochalcone A: a potential multitarget drug for Alzheimer's disease treatment. Int. J. Mol. Sci. 24 (18), 14177. 10.3390/ijms241814177 37762479 PMC10531537

[B31] Puaratanaarunkon T. A.-O. Asawanonda P. A.-O. A. (2022). A randomized, double blinded, split-face study of the efficacy of using a broad spectrum sunscreen with anti-inflammatory agent to reduce post inflammatory hyperpigmentation after Picosecond laser. Clin. Cosmet. Investig. Dermatol 15, 331–337. 10.2147/CCID.S355329 35250287 PMC8894080

[B32] Qu J. Yan M. Fang Y. Zhao J. Xu T. Liu F. (2023). Zebrafish in dermatology: a comprehensive review of their role in investigating abnormal skin pigmentation mechanisms. Front. Physiol. 14, 1296046. 10.3389/fphys.2023.1296046 38074315 PMC10702362

[B33] Reynolds R. V. Yeung H. Cheng C. E. Cook-Bolden F. Desai S. R. Druby K. M. (2024). Guidelines of care for the management of acne vulgaris. J. Am. Acad. Dermatol 90 (5), 1006.e1–1006.e30. 10.1016/j.jaad.2023.12.017 38300170

[B34] Schoelermann A. M. Weber T. M. Arrowitz C. Rizer R. L. Qian K. Babcock M. (2016). Skin compatibility and efficacy of a cosmetic skin care regimen with licochalcone A and 4-t-butylcyclohexanol in patients with rosacea subtype I. J. Eur. Acad. Dermatol Venereol. 30 (Suppl. 1), 21–27. 10.1111/jdv.13531 26805419

[B35] Sharma A. Kroumpouzos G. Kassir M. Galadari H. A.-O. Goren A. A.-O. Grabbe S. (2022). Rosacea management: a comprehensive review. J. Cosmet. Dermatol 21 (5), 1895–1904. 10.1111/jocd.14816 35104917

[B36] Sherwani F. Fau - Nawab M. Nawab M. (2024). Exploring the potential of asl-us-soos (Glycyrrhiza glabra L.) in the treatment of respiratory Diseases-An ethnomedicinal and pharmacological review. Altern. Ther. Health Med. 30 (8), 52–59. 39110056

[B37] ShU J. Cui X. Liu X. Yu W. Zhang W. Huo X. (2022). Licochalcone A inhibits IgE-mediated allergic reaction through PLC/ERK/STAT3 pathway. Int. J. Immunopathol. Pharmacol. 36, 3946320221135462. 10.1177/03946320221135462 36263976 PMC9597022

[B38] Song N. R. Kim J. E. Park J. S. Kim J. R. Kang H. Lee E. (2015). Licochalcone A, a polyphenol present in licorice, suppresses UV-induced COX-2 expression by targeting PI3K, MEK1, and B-Raf. Int. J. Mol. Sci. 16 (3), 4453–4470. 10.3390/ijms16034453 25710724 PMC4394430

[B39] SulzbergeR M. Worthmann A. C. Holtzmann U. Buck B. Jung K. A. Schoelermann A. M. (2016). Effective treatment for sensitive skin: 4-t-butylcyclohexanol and licochalcone A. J. Eur. Acad. Dermatol Venereol. 30 (Suppl. 1), 9–17. 10.1111/jdv.13529 26805417

[B40] Sun C. Wang L. Zhou B. Jiang X. Li H. Liu Z. (2025). Efficient extraction of Licochalcone a with deep eutectic solvent: a promising drug for the treatment of dermatophytosis. Bioorg Chem. 160, 108463. 10.1016/j.bioorg.2025.108463 40233670

[B41] Tang X. Yang T. Yu D. Xiong H. Zhang S. (2024). Current insights and future perspectives of ultraviolet radiation (UV) exposure: friends and foes to the skin and beyond the skin. Environ. Int. 185, 108535. 10.1016/j.envint.2024.108535 38428192

[B42] Tian Y. Luan J. Wang Q. Li C. Peng X. Jiang N. (2024). Licochalcone A ameliorates Aspergillus fumigatus Keratitis by reducing fungal load and activating the Nrf2/HO-1 signaling pathway. ACS Infect. Dis. 10 (10), 3516–3527. 10.1021/acsinfecdis.4c00123 39283729

[B43] Trakatelli M. A.-O. Richard M. A.-O. Rouillard A. Paul C. RöCKEN M. Stratigos A. (2023). The burden of skin disease in Europe. J. Eur. Acad. Dermatol Venereol. 37 (Suppl. 7), 3–5. 10.1111/jdv.19390 37806000

[B44] Udompataikul M. Srisatwaja W. (2011). Comparative trial of moisturizer containing licochalcone A vs. hydrocortisone lotion in the treatment of childhood atopic dermatitis: a pilot study. J. Eur. Acad. Dermatol Venereol. 25 (6), 660–665. 10.1111/j.1468-3083.2010.03845.x 20840345

[B45] Vasam M. Korutla S. Bohara R. A. (2023). Acne vulgaris: a review of the pathophysiology, treatment, and recent nanotechnology based advances. Biochem. Biophys. Rep. 36, 101578. 10.1016/j.bbrep.2023.101578 38076662 PMC10709101

[B46] Wananukul S. Chatproedprai S Fau - Chunharas A. Chunharas A Fau - Limpongsanuruk W. Limpongsanuruk W Fau - Singalavanija S. Singalavanija S Fau - Nitiyarom R. Nitiyarom R Fau - Wisuthsarewong W. (2023). Randomized, double-blind, split-side, comparison study of moisturizer containing licochalcone A and 1% hydrocortisone in the treatment of childhood atopic dermatitis. J. Med. Assoc. Thai 96 (9), 1135–1142. 24163988

[B47] WanG Z. Xue Y. Chen T. Du Q. Zhu Z. Wang Y. (2021). Glycyrrhiza acid micelles loaded with licochalcone A for topical delivery: Co-penetration and anti-melanogenic effect. Eur. J. Pharm. Sci. 167, 106029. 10.1016/j.ejps.2021.106029 34601069

[B48] Wang X. Zhang Z. A.-O. Wang Y. Wu Y. Miao L. Ma Y. (2024). Enrichment of total flavonoids and Licochalcone A from Glycyrrhiza inflata bat. Residue based on a combined membrane-macroporous resin process and a quality-control study. Molecules 29 (10), 2282. 10.3390/molecules29102282 38792142 PMC11124024

[B49] Wang H. Yu W. Wang T. Fang D. Wang Z. Wang Y. (2025). Therapeutic potential and pharmacological insights of total glucosides of paeony in dermatologic diseases: a comprehensive review. Front. Pharmacol. 15, 1423717. 10.3389/fphar.2024.1423717 39822741 PMC11735457

[B50] Wanitphakdeedecha R. A.-O. Tavechodperathum N. Tantrapornpong P. Suphatsathienkul P. Techapichetvanich T. Eimpunth S. (2020). Acne treatment efficacy of intense pulsed light photodynamic therapy with topical licochalcone A, l-carnitine, and decanediol: a spilt-face, double-blind, randomized controlled trial. J. Cosmet. Dermatol 19 (1), 78–87. 10.1111/jocd.13178 31587493

[B51] White J. White M. P. J. Wickremesekera A. Peng L. Gray C. (2024). The tumour microenvironment, treatment resistance and recurrence in glioblastoma. J. Transl. Med. 22 (1), 540. 10.1186/s12967-024-05301-9 38844944 PMC11155041

[B52] Wongtada C. A.-O. Pewlong P. Asawanonda P. Noppakun N. Pongpamorn P. Paemanee A. (2022). Influence of moisturizer containing licochalcone A, 1,2-decanediol, L-carnitine, and salicylic acid on facial skin lipidome among seborrhea participants. J. Cosmet. Dermatol 21 (12), 7081–7089. 10.1111/jocd.15381 36102580

[B53] Yakupu A. Aimaier R. Yuan B. Chen B. Cheng J. Zhao Y. (2023). The burden of skin and subcutaneous diseases: findings from the global burden of disease study 2019. Front. Public Health 11, 1145513. 10.3389/fpubh.2023.1145513 37139398 PMC10149786

[B54] Yang G. Lee H. E. Yeon S. H. Kang H. C. Cho Y. Y. Lee H. S. (2018). Licochalcone A attenuates acne symptoms mediated by suppression of Nlrp3 inflammasome. Phytother. Res. 32 (12), 2551–2559. 10.1002/ptr.6195 30281174

[B55] Zhang Y. Gao M. Chen L. Zhou L. Bian S. Lv Y. A.-O. (2020). Licochalcone A restrains microphthalmia-associated transcription factor expression and growth by activating autophagy in melanoma cells via miR-142-3p/Rheb/mTOR pathway. Phytother. Res. 34 (2), 349–358. 10.1002/ptr.6525 31793097

